# Sodium Rutin Ameliorates Non-Alcoholic Fatty Liver Disease and Alleviates Insulin Resistance by Promoting Lipophagy

**DOI:** 10.3390/ph19040604

**Published:** 2026-04-09

**Authors:** Xue Zhang, Shuoshuo Li, Ping Zhang, Chenggang Zhang, Zengqiang Yuan

**Affiliations:** 1 School of Life Science, Beijing University of Chinese Medicine, Beijing 100029, China; zx15935868462@163.com (X.Z.); 202202002@bucm.edu.cn (S.L.); 2 The Brain Science Center, Beijing Institute of Basic Medical Sciences, Beijing 100850, China; zp15098359497@163.com

**Keywords:** sodium rutin, autophagy, lipophagy, insulin resistance, non-alcoholic fatty liver disease, hepatic steatosis

## Abstract

**Background/Objectives**: Non-alcoholic fatty liver disease (NAFLD) is a prevalent metabolic disorder for which there are limited pharmacotherapies. Sodium rutin (NaR), a soluble flavonoid derivative, has shown beneficial metabolic effects, but its role in NAFLD remains unclear. This study investigates whether NaR ameliorates high-fat diet (HFD)-induced NAFLD and insulin resistance through promoting hepatic lipophagy. **Methods**: Male mice aged 8 weeks old were fed a HFD for 12 weeks with/without NaR supplementation. Body weight was measured every week. After 12 weeks of treatment, GTT and ITT were performed to assess insulin resistance. Then, the tissues were collected and hepatic histology, serum biochemistry, and markers of autophagy and senescence were assessed. **Results**: NaR treatment significantly attenuated HFD-induced weight gain, reduced visceral fat and liver weights, and ameliorated hepatic steatosis and vacuolization. NaR improved serum lipid profiles; lowered alanine aminotransferase, aspartate aminotransferase, and alkaline phosphatase levels; and reduced hepatic cellular senescence. NaR enhanced hepatic autophagy, evidenced by decreased p62 levels, increased LC3-II/LC3-I ratio, and enhanced colocalization of lipid droplets with LC3 and LAMP1 in vivo and in vitro. These changes were accompanied by improved glucose tolerance and insulin sensitivity. **Conclusions**: NaR effectively alleviates HFD-induced NAFLD and insulin resistance by activating hepatic lipophagy. These findings support NaR as a promising multi-targeted therapeutic candidate for NAFLD.

## 1. Introduction

Non-alcoholic fatty liver disease (NAFLD) is a progressive hepatic disorder characterized by excessive lipid deposition in hepatocytes in the absence of significant alcohol intake. The disease spectrum spans from simple steatosis to non-alcoholic steatohepatitis, which may progress to cirrhosis and hepatocellular carcinoma [1,2]. The global prevalence of NAFLD has risen in parallel with the increasing incidence of obesity and type 2 diabetes. Despite this trend, the precise pathological mechanisms remain incompletely understood, and no pharmacological therapies are currently approved for this condition [3,4].

Insulin resistance and hepatic lipid accumulation are central features of NAFLD. During disease progression, insulin resistance in adipose tissue enhances lipolysis, leading to an increased flux of free fatty acids to the liver, which subsequently promotes hepatic steatosis and dysfunction [5,6,7,8]. Cellular senescence, a state of sustained cell-cycle arrest, has garnered significant attention as a potential contributor to the development of metabolic diseases, particularly NAFLD and cirrhosis [9]. More importantly, lipid accumulation exacerbates hepatic senescence [10]. Senescence may also predispose adipose tissue to insulin resistance [11]. Our previous study demonstrated that sodium rutin (NaR), a soluble form of rutin, ameliorates hepatic steatosis during the aging process, primarily by enhancing autophagy and promoting lipid oxidation [12,13]. In vivo studies demonstrated that rutin attenuates hepatotoxicity in rats on a high-cholesterol diet [14]. The antioxidant activity of rutin can be enhanced through lipase-mediated esterification with oleic acid (OA) [15]. Furthermore, rutin exhibits hepatoprotective effects in a mouse model of NAFLD by reducing hepatic lipid levels and mitigating lipid-induced oxidative injury [16]. Interestingly, rutin has been shown to attenuate lipid accumulation by decreasing lipogenesis and alleviating oxidative stress in hepatocytes, while increasing AMPK activity [17]. However, the hepatoprotective mechanism of NaR in NAFLD remains elusive. Recent research has confirmed that rutin ameliorates lipid metabolism in diabetic NAFLD by activating the AMPK pathway [18]. These findings provide important evidence for further elucidating the mechanism underlying the therapeutic effects of NaR on NAFLD. Whether NaR exerts its therapeutic effects against NAFLD through mechanisms beyond the AMPK pathway remains to be explored. Restoring autophagic flux, either pharmacologically or genetically, ameliorates hepatic steatosis in experimental models [19,20,21]. Therefore, we hypothesized that NaR confers therapeutic benefits in NAFLD by promoting autophagy.

Accordingly, this study aims to investigate the effects of NaR on NAFLD using a high-fat diet (HFD)-induced mouse model. We also aim to investigate whether its protective effects are mediated by promoting hepatic lipophagy and alleviating systemic insulin resistance. The results demonstrate that NaR effectively attenuates liver injury and steatosis, improves insulin sensitivity, and enhances autophagic activity, thereby positioning it as a promising therapeutic candidate for NAFLD.

## 2. Results

### 2.1. NaR Treatment Alleviates HFD-Induced Obesity and Hepatic Steatosis

**Figure 1:**
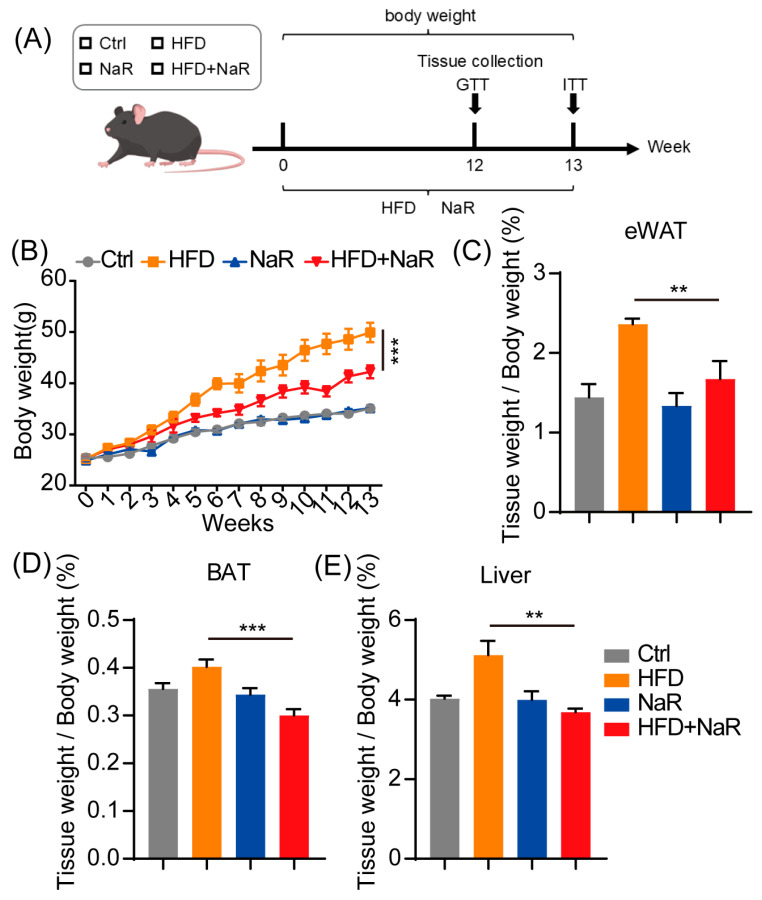
NaR alleviates HFD-induced obesity. (**A**) The flow chart of the experiments. (**B**) Body weight changes in mice on normal or HFD over 12 weeks. (**C**) Ratio of eWAT weight to body weight after 12 weeks of normal or HFD. (**D**) Ratio of BAT weight to body weight. (**E**) Ratio of liver weight to body weight (B: two-way ANOVA; others: Student’s *t*-test. ** *p* < 0.01, *** *p* < 0.001).

NaR administration exerted no effect on body weight in mice fed a normal diet, whereas it markedly attenuated HFD-induced weight gain over 12 weeks (Figure 1A,B). Moreover, NaR supplementation significantly reduced epididymal white adipose tissue (eWAT), brown adipose tissue (BAT), and liver weights in HFD-fed mice (Figure 1C–E), indicating that NaR alleviates HFD-induced hepatic steatosis and adiposity.

**Figure 2:**
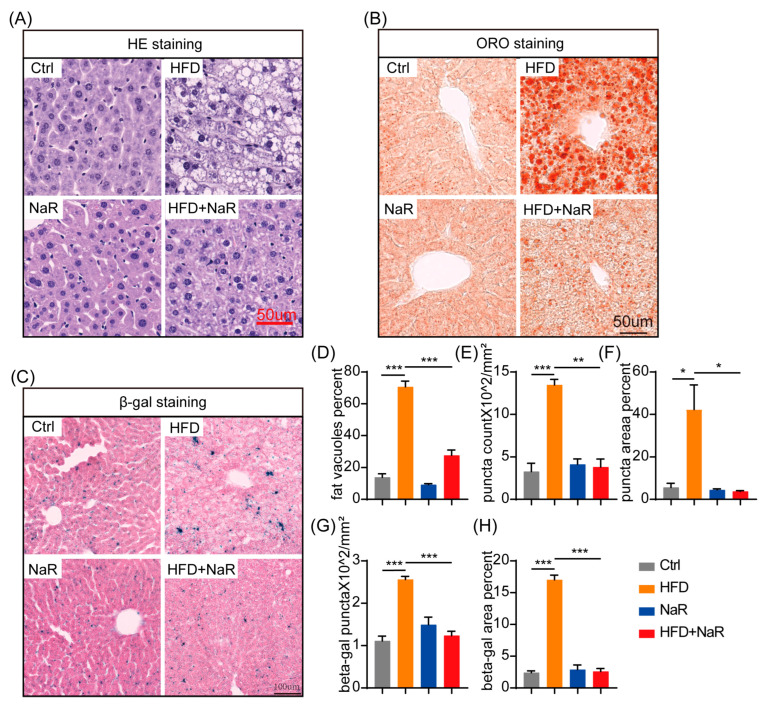
NaR attenuates hepatic steatosis and cellular senescence in HFD-fed mice. (**A**–**C**) Representative images of liver sections stained with H&E, Oil Red O and β-gal following 12 weeks of HFD feeding. (**D**) Statistics of the number of hepatic fat vacuoles. (**E**,**F**) Statistics of the number and area of hepatic LDs. (**G**,**H**) Statistics of the number and area of hepatic β-gal positive spots (Student’s *t*-test, * *p* < 0.05, ** *p* < 0.01, *** *p* < 0.001).

Histopathological analysis further confirmed these findings. Hematoxylin and eosin (H&E) staining revealed marked hepatic vacuolization in HFD-fed mice, which was significantly ameliorated by NaR treatment (Figure 2A,D). Correspondingly, Oil Red O staining showed that NaR markedly reduced both the number and size of lipid droplets (LDs) in the liver (Figure 2B,E,F). Collectively, these data demonstrate that NaR effectively ameliorates HFD-induced obesity and hepatic steatosis.

### 2.2. NaR Ameliorates Hepatic Injury and Reduces Cellular Senescence in HFD-Fed Mice

**Figure 3:**
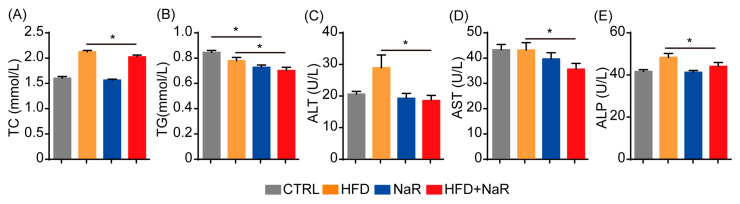
NaR improves metabolic parameters and attenuates liver injury in HFD-fed mice. (**A**) Measurement of serum TG levels following 12 weeks of HFD induction. (**B**) Measurement of serum TC levels following 12 weeks of HFD induction. (**C**) Measurement of serum ALT levels following 12 weeks of HFD induction. (**D**) Measurement of serum AST levels following 12 weeks of HFD induction. (**E**) Measurement of serum ALP levels following 12 weeks of HFD induction (Student’s *t*-test, * *p* < 0.05).

To assess the impact of NaR on hepatic function and injury, serum biochemical analyses were performed. NaR treatment significantly reduced serum total cholesterol (TC) and triglyceride (TG) levels in HFD-fed mice (Figure 3A,B). Furthermore, key liver injury markers—alanine aminotransferase (ALT), aspartate aminotransferase (AST), and alkaline phosphatase (ALP)—were significantly decreased with NaR treatment (Figure 3C–E).

As cellular senescence is closely associated with metabolic dysfunction and liver injury, we also evaluated senescent cell accumulation using β-galactosidase (β-gal) staining. NaR treatment significantly reduced both the number and area of β-gal-positive spots in the liver (Figure 2C,G,H), suggesting mitigation of HFD-induced cellular senescence. Collectively, these results demonstrate that NaR not only mitigates hepatic lipid accumulation but also attenuates liver injury and senescence in HFD-induced NAFLD.

### 2.3. NaR Promotes Hepatic Lipophagy Under HFD Conditions

**Figure 4:**
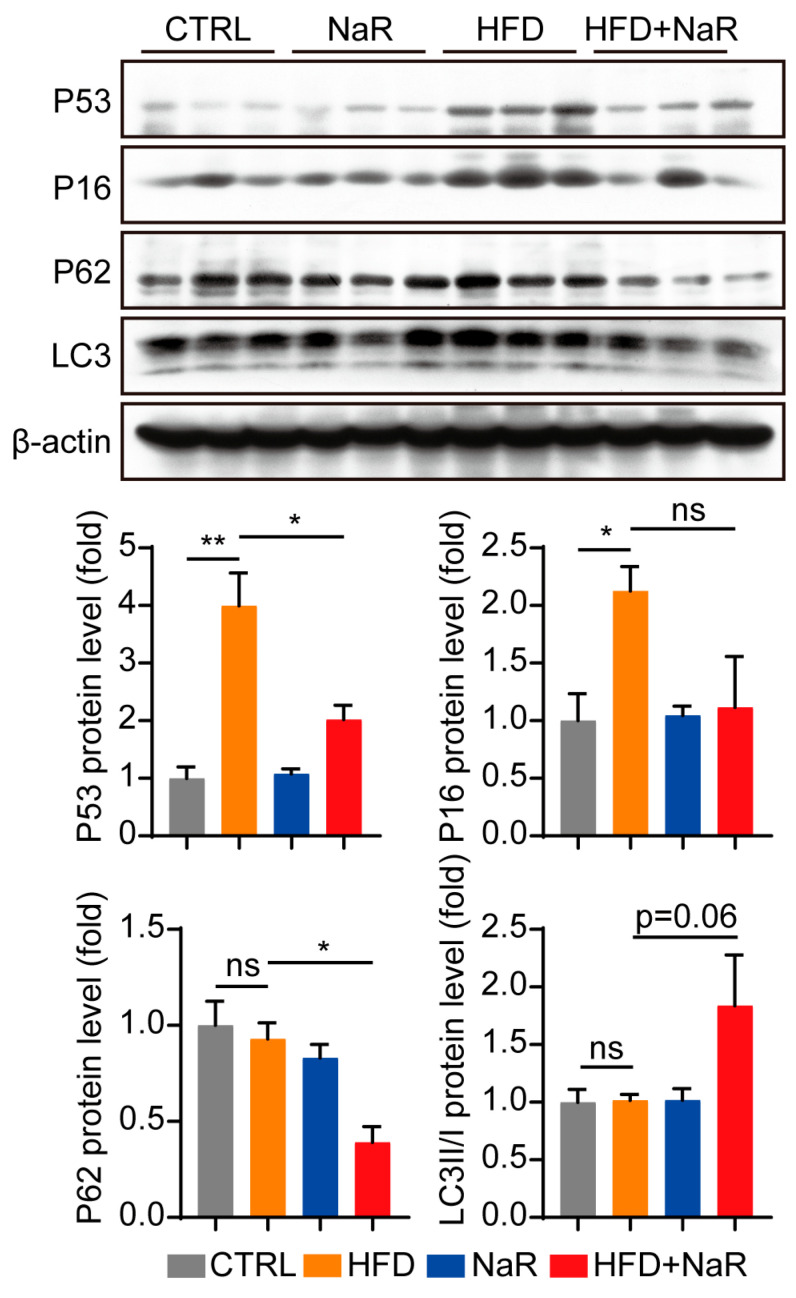
NaR improves hepatocytes autophagy level in HFD conditions. Mice were subjected to a HFD and/or NaR feeding, followed by analysis of the protein expression levels of P53, P16, P62, and LC3 (Student’s *t*-test. * *p* < 0.05, ** *p* < 0.01, “ns” indicates no significant difference).

Given that autophagy plays a critical role in hepatic lipid metabolism, we next investigated whether NaR exerts its hepatoprotective effects through the activation of lipophagy. In vivo, NaR treatment significantly downregulated p62 protein levels and increased the LC3-II/LC3-I ratio in the liver of HFD-fed mice (Figure 4). Concomitantly, NaR also downregulated senescence-related proteins p53 and p16, suggesting a link between enhanced autophagy and reduced cellular senescence (Figure 4).

**Figure 5:**
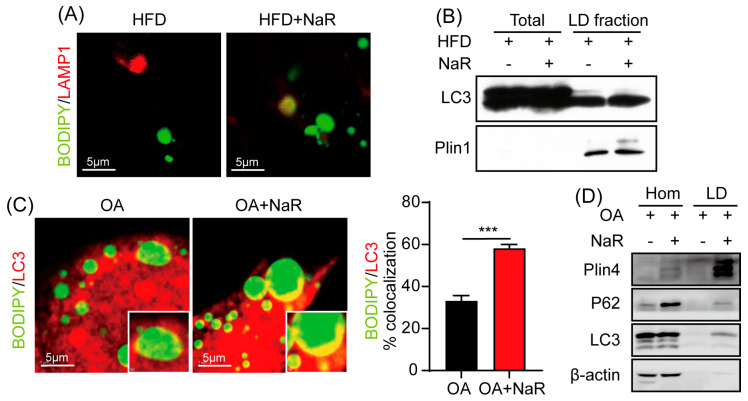
Both in vivo and in vitro, NaR enhances hepatic autophagy under HFD conditions. (**A**) Representative and quantitative immunofluorescence images of early autophagosomes (yellow puncta, BODIPY colocalized with LAMP1) and autolysosomes (red puncta) in liver sections from mice fed a HFD with or without NaR treatment. (**B**) Western blot analysis of Plin1 and LC3 protein expression in LDs isolated from the livers of mice fed a HFD with or without NaR treatment. (**C**) Representative and quantitative image of early autophagosomes (yellow foci, BODIPY colocalized with LC3), LDs (green foci) and autolysosomes (red foci, LC3) in HepG2 cells. (**D**) Western blot analysis of Plin4, P62, and LC3 protein expression following lipid droplet isolation from HepG2 cells (Student’s *t*-test. *** *p* < 0.001).

To further assess autophagic activity in the liver, we used immunofluorescence to stain for LAMP1, a marker of autolysosomes. NaR treatment significantly increased the colocalization of LDs with LAMP1 in hepatic sections (Figure 5A), indicating increased autophagic activity under high-fat conditions. Western blot analysis of isolated hepatic LDs further supported this finding, demonstrating increased LC3B levels following NaR treatment (Figure 5B).

In parallel, we conducted in vitro experiments to validate these findings. To establish a cellular model of NAFLD, HepG2 cells were treated with OA. Immunofluorescence staining revealed a significant increase in the colocalization of LDs and LC3 upon NaR treatment, indicating that NaR promoted autophagy in HepG2 cells under high-lipid conditions (Figure 5C). Furthermore, LDs were isolated from HepG2 cells treated with both OA and NaR. Western blot analysis of isolated LDs showed increased LC3B levels after NaR treatment, consistent with the in vivo findings in mice (Figure 5D). These consistent in vivo and in vitro results demonstrate that NaR robustly activates hepatic lipophagy under conditions of lipid overload.

### 2.4. NaR Attenuates HFD-Induced Insulin Resistance

**Figure 6:**
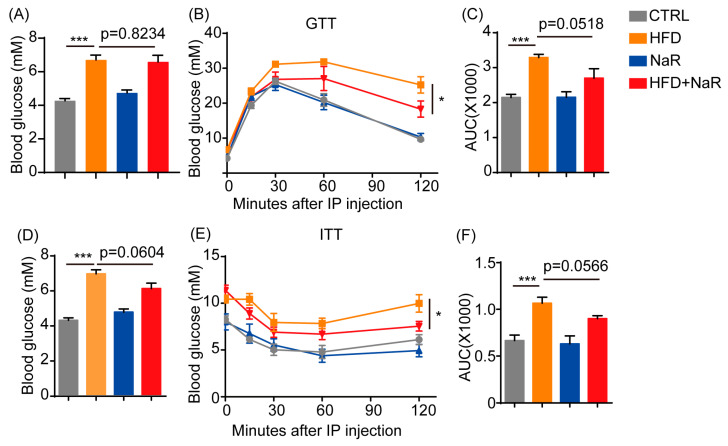
NaR treatment alleviates HFD-induced insulin resistance. (**A**) Fasting blood glucose levels after a 16-h fast. (**B**) GTT after 12 weeks of HFD. (**C**) Quantification of GTT after 12 weeks of HFD. (**D**) Fasting blood glucose levels after a 4-h fast. (**E**) ITT after 13 weeks of HFD. (**F**) Quantification of ITT after 13 weeks of HFD (C, F: two-way ANOVA; others: Student’s *t*-test. * *p* < 0.05, *** *p* < 0.001).

Adipose tissue plays a pivotal role in insulin sensitivity, and HFD-induced liver injury is often accompanied by insulin resistance [22]. Enhanced hepatic autophagy has been linked to improved systemic insulin sensitivity. Therefore, we investigated whether NaR-induced lipophagy mediates metabolic improvement in HFD-fed mice. After 12 weeks of HFD feeding, NaR-treated mice exhibited significantly lower fasting blood glucose levels following both 16-h and 4-h fasts (Figure 6A,D). Glucose tolerance test (GTT) revealed that NaR ameliorated HFD-induced glucose intolerance, as evidenced by a more rapid return to baseline glucose levels (Figure 6B,C). Similarly, insulin tolerance test (ITT) showed that NaR improved insulin sensitivity, with accelerated glucose disposal following insulin injection (Figure 6E,F).

Notably, these metabolic improvements were observed specifically in HFD-fed mice, with no significant effects under normal dietary conditions. Together, these findings demonstrate that NaR effectively alleviates HFD-induced insulin resistance, likely mediated, at least in part, by hepatic lipophagy.

## 3. Discussion

Obesity, characterized by excessive adipose tissue accumulation, is a major risk factor for metabolic syndrome and associated complications [23,24]. Previous work from our laboratory demonstrated that NaR treatment ameliorates hepatic lipid accumulation in mice, promoting a shift toward fatty acid oxidation as the primary energy source [13]. In the present study, NaR treatment effectively alleviated HFD-induced obesity, which is consistent with our previous findings in aging mice. Interestingly, NaR specifically counteracted HFD-induced weight gain and visceral adiposity without affecting normal growth, suggesting it may selectively target pathways involved in metabolic regulation. The reduction in eWAT, BAT, and liver weights, along with histological improvement, corroborates the efficacy of NaR in alleviating hepatic steatosis and associated pathology. Furthermore, NaR treatment led to significant improvements in systemic lipid profiles and liver injury markers, along with histological recovery and a reduction in cellular senescence. Our results indicate that NaR treatment alleviates HFD-induced lipid accumulation and liver injury, corroborating previous findings [16,17,18].

Dysfunctional adipose tissue in obesity plays a pivotal role in the development of insulin resistance [25,26,27]. Insulin resistance, characterized by diminished responsiveness of peripheral tissues to insulin, impairs glucose homeostasis and constitutes a key pathological basis for obesity-associated disorders, including type 2 diabetes, cardiovascular diseases, and other chronic conditions [28,29]. Given the established link between adipose tissue dysfunction and insulin resistance, we evaluated NaR’s impact on glucose homeostasis. NaR improved glucose tolerance, insulin sensitivity, and fasting glycemia, highlighting its role as an insulin-sensitizing agent likely through multi-tissue regulation.

Our findings are largely consistent with those reported by Liu et al. [18] who demonstrated that rutin ameliorated lipid metabolism dysfunction in a diabetic NAFLD model by activating the AMPK/SREBP1 pathway. While their study focused on diabetic NAFLD, our work investigates NAFLD in a broader context, thereby expanding the potential therapeutic scope of the intervention. Furthermore, Liu et al. [18] attributed the effects of rutin primarily to AMPK pathway activation, whereas our study identifies hepatic lipophagy as a key downstream mechanism. As AMPK is a well-established regulator of autophagy, it is suggested that NaR may exert its effects through AMPK-dependent activation of lipophagy. This suggests that AMPK activation and lipophagy may share the same mechanism rather than being contradictory.

Several studies have linked the function and regulation of autophagy with cellular lipid metabolism [30,31]. While a prior study reported the lipid-lowering effect of NaR [17], its underlying cellular mechanism remained undefined. Of note, one study reported that rutin suppressed autophagic function in the liver by downregulating tumor necrosis factor-alpha and interleukin-1 beta, thereby promoting fatty acid metabolism and inhibiting lipogenesis [16]. This finding appears to contradict our observation that NaR activates autophagic flux under lipid overload. It should be noted that the models and chemical dosages used are different, and that rutin may have context-specific effects. Additionally, diet-induced or genetic obesity has been shown to decrease autophagic function in the liver [32,33]. In contrast, our study reveals a distinct pathway by demonstrating that NaR robustly activates autophagic flux under lipid overload. The in vivo reduction in p62 and the increase in the LC3-II/LC3-I ratio, coupled with in vitro evidence of enhanced lipid droplet-LC3 colocalization and increased LC3B in isolated LDs, firmly establish the activation of lipophagy as a core mechanism. These findings align with a growing body of evidence indicating that pharmacological activation of autophagy represents a viable therapeutic strategy for NAFLD [19,21]. Moreover, the concomitant downregulation of p53 and p16 suggests a mechanistic link between NaR-induced autophagy and its anti-senescence effects, consistent with previous reports linking autophagic dysfunction to cellular senescence in NAFLD progression [9].

In conclusion, NaR exerts comprehensive therapeutic effects against NAFLD and associated metabolic disorders by coordinately regulating lipid metabolism, insulin sensitivity, autophagic activity, and cellular senescence. This study provides strong preclinical evidence for NaR as a promising multi-targeted agent for NAFLD, offering a solid foundation for its future clinical development. It should be noted that this study was conducted primarily in male mice; future investigations should evaluate potential sex differences in NaR’s efficacy.

## 4. Materials and Methods

### 4.1. Animal Housing and Experimental Conditions

Animal housing: All mice used in this study were male C57BL/6J mice. Mice were housed under a 12 h light/dark cycle (lights on from 8: 00 to 22: 00) in a specific pathogen-free facility. The temperature was 22 ± 2 °C, and the humidity was 50 ± 10%.

Animal model and chemical treatment: The experimental animals were divided into 4 groups: Ctrl, HFD, NaR, HFD+NaR. To establish the chronic liver injury model, 8-week-old mice were fed a HFD (containing 60% fat, D12492, SIPEIFU, Beijing, China) for 12 weeks, whereas control group mice were fed a standard mouse breeding diet (SPF-F02-002, SIPEIFU, Beijing, China). In NaR treatment groups, mice had free access to the water supplemented with NaR at a final concentration of 0.2 mg·mL^−1^ as previously reported [12,13], whereas other groups had access to the normal water.

Duration, endpoint and ethics: 8-week-old mice were fed a HFD for 12 weeks to establish a model of chronic liver damage. After 12 weeks of treatment, the body weight showed a significant difference; therefore, the mice were subjected to sample collection following GTT and ITT experiments. All animal studies were conducted in accordance with protocols approved by the Institutional Animal Care and Use Committee of the Beijing Institute of Basic Medical Sciences (IACUC-DWZX-2019-504) (Beijing, China).

### 4.2. GTT and ITT

For the GTT, mice were fasted for 16 h prior to the test but maintained free access to drinking water. Following intraperitoneal injection of glucose (2 g·kg^−1^, 10010518, SCR, Shanghai, China), blood glucose levels were measured at 0, 15, 30, 60, and 120 min using a glucometer. For the ITT, mice were fasted for 4 h prior to the test while maintaining free access to drinking water. Following intraperitoneal injection of insulin (0.75 U·kg^−1^, 10908, Sigma Aldrich, St. Louis, MO, USA), blood glucose levels were measured at 0, 15, 30, 60, and 120 min using a glucometer (582, yuwell, Danyang, China).

### 4.3. Tissue Collection

Each mouse was weighed prior to tissue collection. After the mice were anesthetized with tribromoethanol (A0415706, Thermo, Waltham, MA, USA), blood was collected from the right ventricle. Serum was obtained by centrifuging the blood at 3000× *g* for 10 min. Then, liver tissues were collected. eWAT was carefully dissected from the testes and epididymis in male mice, and interscapular BAT was harvested from the interscapular region. All collected tissues were weighed immediately.

### 4.4. Histology

Liver tissues from mice were embedded in paraffin and sectioned at a thickness of 4 μm for H&E staining (hematoxylin, 0701 and eosin, 0109, AMRESCO, Solon, OH, USA). Additionally, frozen liver sections were prepared at 20 μm and stained with Oil Red O (O0625, Sigma Aldrich, St. Louis, MO, USA) and β-gal (C0602, Beyotime, Shanghai, China).

### 4.5. Serum Biochemistry

After 12 weeks of a HFD, serum samples were collected from the mice for various tests. TG and TC levels were measured using commercial measurement kits (E1013 and E1015, Applygen, Beijing, China) according to the manufacturer’s instructions. ALT, AST, and ALP levels were measured using a blood chemistry analyzer (BS-240VET, Mindray, Shenzhen, China).

### 4.6. Cell Culture

The cell lines used in this study is HepG2 that was obtained from ATCC bioresource center. The HepG2 cell line was cultured in Dulbecco’s Modified Eagle Medium (DMEM; C11995500BT, Gibco, Waltham, MA, USA) supplemented with 10% fetal bovine serum (FBS; C04001-500, Viva Cell BIOSCIENCES, Shanghai, China) and 1% penicillin–streptomycin solution (C3420-0100, Viva Cell BIOSCIENCES, Shanghai, China). Cells were cultured at 37 °C in a humidified atmosphere containing 5% CO_2_. At the indicated time points, the following compounds were added to the culture medium: NaR (200 μM for 24 h) and OA (500 μM for 12 h, S4707, Selleckchem, Houston, TX, USA).

### 4.7. Isolation and Purification of LDs from Cell Lines

LDs were isolated from cultured cells via density gradient ultracentrifugation. Briefly, cell pellets that were collected and washed with PBS were resuspended in ice-cold Buffer A (20 mM Tricine, 250 mM sucrose, pH 7.8, containing 0.2 mM PMSF) and incubated on ice for 15 min to facilitate cell lysis. The homogenate was then centrifuged at 1000× *g* for 10 min at 4 °C to remove nuclei and cellular debris. The resulting post-nuclear supernatant was collected, and a 500 μL aliquot was saved for subsequent purity analysis. The remaining post-nuclear supernatant was transferred to a new tube, overlaid with a one-fifth volume of Buffer B (20 mM HEPES, 100 mM KCl, 2 mM MgCl_2_, pH 7.4), and subjected to ultracentrifugation at 20,630× *g* for 1 h at 4 °C. The LDs, which floated to the top as a milky layer, were carefully collected using pre-wetted pipette tips, transferred to a 1.5 mL tube, and washed three times with Buffer B. The purified LD fraction was finally analyzed by Western blotting.

### 4.8. Immunofluorescence Staining

HepG2 cells were seeded on glass coverslips. Following treatments with OA and NaR, cells were fixed with 4% paraformaldehyde, permeabilized with PBST, and blocked with blocking buffer for 1 h. Subsequently, cells were incubated overnight at 4 °C with primary antibody against LC3 (1:200, L7543, Sigma Aldrich, St. Louis, MO, USA). The following day, samples were incubated with Alexa Fluor 568-conjugated secondary antibody (A-11004, Invitrogen, Carlsbad, CA, USA) for 1 h, followed by staining with BODIPY (D3922, Thermo, Waltham, MA, USA) and DAPI (D9542, Sigma Aldrich, St. Louis, MO, USA) to visualize LDs and nuclei, respectively. In liver tissue, LAMP1 (1:200, AB2971, Sigma Aldrich, St. Louis, MO, USA) serves as a marker for autophagic lysosomes. Photographed with confocal microscope (nikonA1, Tokyo, Japan).

### 4.9. Immunoblotting

Tissues or cells were lysed in RIPA buffer containing a protease and phosphatase inhibitor cocktail. Protein concentration was determined using a BCA protein assay kit (P0009-1, Beyotime, Shanghai, China), followed by boiling the samples in 6 × sodium dodecyl sulfate loading buffer. Proteins were separated by sodium dodecyl sulfate-polyacrylamide gel electrophoresis and electrophoretically transferred onto nitrocellulose membranes (GE Amersham, 10600002, Sigma Aldrich, St. Louis, MO, USA). The membranes were then blocked with 5% non-fat milk, followed by incubation with primary antibodies at 4 °C overnight. Subsequently, the membranes were incubated with HRP-conjugated secondary antibodies for protein detection. The following primary antibodies were used: anti-LC3 (1:1000, 14600-1-AP, Proteintech, Rosemont, IL, USA), anti-p62 (1:1000, F0106, Selleckchem, Houston, TX, USA), anti-Plin4 (1:1000, AB234752, Abcam, Cambridge, UK), anti-p53 (1:1000, Sc126, Santa cruz, Texas, USA), anti-p16 (1:1000, AB51243, Abcam, Cambridge, UK), anti-Plin1 (1:1000, AB3526, Abcam, Cambridge, UK),and anti-β-actin (1:10,000, Proteintech, Rosemont, IL, USA). Secondary antibodies included HRP-conjugated goat anti-rabbit (1:5000, 115-035-003, Jackson, San Bernardino, CA, USA) and HRP-conjugated goat anti-mouse (1:5000, 115-035-003, Jackson, San Bernardino, CA, USA). Protein band intensities were quantified by densitometry and normalized to the corresponding β-actin loading control.

## 5. Conclusions

This study demonstrates that NaR treatment ameliorates HFD-induced obesity, hepatic steatosis, and insulin resistance by promoting hepatic lipophagy. These findings highlight NaR as a promising multi-targeted candidate for the treatment of NAFLD.

## Data Availability

The original contributions presented in this study are included in the article. Further inquiries can be directed to the corresponding authors.

## Abbreviations

The following abbreviations are used in this manuscript:

NAFLD	Non-alcoholic fatty liver disease
NaR	Sodium rutin
HFD	High-fat diet
ALT	Alanine aminotransferase
AST	Aspartate aminotransferase
ALP	Alkaline phosphatase
AMPK	Adenosine 5′-monophosphate-activated protein kinase
H&E	Hematoxylin and eosin
β-gal	β-galactosidase
eWAT	Epididymal white adipose tissue
BAT	Brown adipose tissue
TG	Triglyceride
TC	Total cholesterol
GTT	Glucose tolerance test
ITT	Insulin tolerance test
LDs	Lipid droplets
LC3	Microtubule-associated protein 1 light chain 3
P62	Sequestosome 1
Plin1	Perilipin 1
Plin4	Perilipin 4
OA	Oleic acid
